# Comparative Genomic Analyses of *E. coli* ST2178 Strains Originated from Wild Birds in Pakistan

**DOI:** 10.4014/jmb.2407.07026

**Published:** 2024-08-29

**Authors:** Jung Hun Lee, Abdul Rauf Tareen, Nam-Hoon Kim, Chanyeong Jeong, Byeonghyeon Kang, Gwangje Lee, Dae-Wi Kim, Rabaab Zahra, Sang Hee Lee

**Affiliations:** 1 National Leading Research Laboratory of Drug Resistance Proteomics, Department of Biological Sciences, Myongji University, Yongin 17058, Republic of Korea; 2 Department of Microbiology, Quaid-i-Azam University, Islamabad 45320, Pakistan; 3 Department of Life Sciences, Jeonbuk National University, Jeonju 54896, Republic of Korea

**Keywords:** *Escherichia coli*, ST2178, plasmid, antibiotic resistance gene, virulence factor, serotype

## Abstract

The emergence and spread of multidrug-resistance (MDR) pathogenic *Escherichia coli* due to horizontal gene transfer of antibiotic resistance genes (ARGs) and virulence factors (VFs) is a global health concern, particularly in developing countries. While numerous studies have focused on major sequence types (STs), the implication of minor STs in ARG dissemination and their pathogenicity remains crucial. In this study, two *E. coli* strains (PEC1011 and PEC1012) were isolated from wild bird feces in Pakistan and identified as ST2178 based on their complete genome sequences. To understand this minor ST, 204 genome assemblies of ST2178 were comparatively analyzed with the isolates’ genomes. The phylogenetic analyses revealed five subclades of ST2178. Subclade E strains were predominantly isolated from human specimens, whereas subclades A and B strains including strains PEC1011 and PEC1012, respectively, were frequently isolated from animal. Mobile genetic elements (MGEs) exhibited the positive correlation with ARGs but not with VFs in this ST. Plasmid-borne ARGs exhibited higher correlation with plasmid-borne MGEs, indicating the role of diverse mobile plasmid structures in ARG transmission. Subclade E exhibited diverse plasmid-borne ARG repertoires correlated with MGEs, marking it as a critical surveillance target. In the case of VFs, they exhibited phylogeny-dependent profiles. Strain PEC1012 harbored various plasmid-borne ARGs, which are similar with conserved ARG repertoires in subclade A. The presence of unique ARG insertion in pPEC1012 highlights the importance of subclade A in ARG dissemination. This study comprehensively elucidates the landscape of ST2178, identifying critical phylogenetic subclades and their characteristics in ARG and VF occurrence.

## Introduction

*Escherichia coli* is a versatile bacterial species present in diverse ecological niches, ranging from the intestinal tracts of humans and animals to various environmental habitats [1]. Although generally recognized as a commensal bacterium of the intestine, *E. coli* is also an opportunistic pathogen responsible for broad range of infections in human and animals [2]. Based on infections sites, pathogenic *E. coli* is classified into two major pathogroups: intestinal pathogenic *E. coli* (InPEC) and extraintestinal pathogenic *E. coli* (ExPEC) [3]. ExPEC encompasses pathotypes such as uropathogenic *E. coli* (UPEC), neonatal meningitis-associated *E. coli* (NMEC), and sepsis-associated *E. coli* (SEPEC). In contrast, InPEC, also referred as diarrheagenic *E. coli* (DEC), includes major types such as enteropathogenic *E. coli* (EPEC), enteroinvasive *E. coli* (EIEC), enterotoxigenic *E. coli* (ETEC), enterohemorrhagic *E. coli* (EHEC), enteroaggregative *E. coli* (EAEC), diffusely-adherent *E. coli* (DAEC), and adherent-invasive *E. coli* (AIEC) [2]. While specific virulence gene markers have been used to define these pathotypes, relying solely on a few genes is limited in its accuracy [2]. Furthermore, genetic plasticity of *E. coli* and its propensity for acquiring virulence factors (VFs) through horizontal gene transfer suggest that pathotypes alone cannot reliably predict the clinical outcomes of infections caused by different *E. coli* strains [3]. These considerations underscore the necessity of thorough genome-level investigation to fully understand the pathogenicity of *E. coli*.

In addition to VFs, antibiotic resistance genes (ARGs) in *E. coli* represent a major health concern due to their capacity to acquire ARGs though horizontal gene transfer. The emergence of *E. coli* strains carrying critical ARGs such as those encoding expanded-spectrum β-lactamases (ESBLs), carbapenemases, 16S rRNA methylases, plasmid-mediated quinolone resistance, and colistin resistance, is becoming increasingly problematic worldwide [4]. Considering the ubiquity of *E. coli* in One-health sectors and their propensity for frequent horizontal exchange of ARGs and VFs, the surveillance of animal- and environment-originated *E. coli* is strongly demanded. Systematic comprehension on these isolates is essential to scrutinize the virulence and antibiotic resistance characteristics [5, 6]. Notably, among animals, wild birds have recently been recognized as carriers of multidrug-resistant (MDR) *E. coli*, posing a risk to human through fecal contamination [7].

To understand the virulence and antibiotic resistance of *E. coli*, comparative analyses based on large-scale genome sets are vital, as they reveal critical ARGs in specific sequence types (ST) [8]. However, the role of minor STs in the dissemination of virulence and antibiotic resistance is often overlooked. This suggests that a systematic understanding of minor ST based on comparative genomics is both pivotal and urgently needed.

The high rate of emergence of ARGs in developing countries presents a major challenge in understanding and mitigating ARG transmission worldwide. In Pakistan, several reports highlight the high prevalence of ARGs and antibiotic resistance bacteria, underscoring the need for a systematic understanding of isolates from the country. The spread and high incidence of ARGs in Pakistan pose regional threats and, eventually, a global menace [9, 10].

In this study, two *E. coli* strains were isolates from wild bird feces in Islamabad, Pakistan, and their complete genome sequences were obtained, revealing they belonged to a minor ST, ST2178. To understand virulence factors and antibiotic resistance, phylogenetic lineages and serotype, comparative genomic analyses were conducted on these isolates and all ST2178 strains available in the public database.

## Materials and Methods

### Isolation of *E. coli* Strains and Complete Genome Sequencing

Fecal samples of wild birds were collected between 2016 and 2017 in Pakistan. *E. coli* strains were isolated from the samples using liquid LB medium with 100 mg/ml ampicillin (Duchefa Biochemie B. V., The Netherlands). The isolates were cultured in liquid LB medium and the cells were harvested to extract genomic DNA. The extraction was performed using the Qiagen MagAttract HMW DNA kit (Qiagen, Germany) according to the manufacture’s instruction. The concentration and quality of the extracted DNA were verified using the Qubit 2.0 fluorometer (Invitrogen, USA) to ensure they met the sequencing criteria of the sequencing service company (CJ Bioscience Inc, Republic of Korea). A hybrid sequencing approach employing MiSeq (Illumina, USA) and PacBio Sequel (Pacific Biosciences, USA) platforms was used to obtain the complete genome sequences. TruSeq DNA Library LT kit (Illumina) and SMRTbell Express Template Preparation Kit were used for construction of libraries for MiSeq and PacBio Sequel sequencing, respectively. Hybrid assembly was conducted using Unicycler version 0.4.9 using quality controlled MiSeq data and PacBio long reads data. Protein-coding sequences (CDSs) were predicted by Prodigal 2.6.2 [11]. Complete genome sequences of *E. coli* strains obtained in this study have been deposited in the GenBank under accession numbers of CP159540 (chromosome of strain PEC1011), CP159541 (plasmid of strain PEC1011), CP161338 (chromosome of strain PEC1012), and CP161339 (plasmid of strain PEC1012).

### Phylogenomic Analyses

To conduct comprehensive comparative genomic analyses, 204 genome assemblies belonging to ST2178 were retrieved from the EnteroBase [12]. The Roary tool (version 3.13.0) was employed to select and calculate core-genes of the genome assemblies, which included the outgroup ST58 (strain MI-12-184; NCBI assembly accession No. GCA_020358995.1) [13]. A total of 3,615 core genes were obtained using a 99% identity cutoff. Alignment of core-genome was performed using the MAFFT [14]. Subsequently, a core-genome phylogenomic tree was constructed using the FastTree (version 2.1.11) using Maximum Likelihood method [15]. The interactive tree of life (iTOL, v5) was used to visualize the core-genome tree along with heatmaps depicting the relative abundance of ARGs, VFs, plasmid replicon sites, and serotypes [16].

### Comparative Genomic Analyses

Multi locus sequence typing (MLST) of the genome assemblies was performed using pubMLST (version 2.23.0)[17] based on the Achtman scheme [18]. ClermonTyping was also employed for phylogroup analyses [19]. Metadata of the genome assemblies such as isolation sources, were also collected from the EnteroBase. Annotation of 206 assemblies were accomplished with Prokka tool (version 1.14.6) with the default setting [20]. Resistance gene identifier (RGI version 6.0.3) was employed to search ARGs in the genome assemblies. The parameters for the RGI analysis were set to include only strict and perfect hits, excluding nudge (https://github.com/arpcard/rgi) [21]. Serotype, virulence factors and plasmid replicon sites were identified by ABRicate with the default settings (version 1.0.1, https://github.com/tseemann/abricate) using each other database ECOH, VFDB, Plasmidfinder (as of Nov. 4^th^ 2023), respectively. FimH (type-1 fimbrial adhesin) type was confirmed using fimtyper with the default settings [22]. MEfinder (version 1.1.2) with the default settings was hired to find mobile genetic elements (MGEs) and the geNomad tool (version 1.7.6) with the default settings was used to find plasmid-borne contigs in the assemblies [23, 24].

### Genetic Structure Analyses of Plasmids

Using the complete pPEC1012 nucleotide sequence, contigs harboring conserved genetic structures of pPEC1012 were searched in four genomes of subclade A strains, which exhibited similar ARG repertoires to those of pPEC1012. Among the hits, ARG-carrying contigs were extracted and compared with pPEC1012 using Clinker tool [25].

## Results and Discussion

### Complete Genome Sequences of Two *E. coli* Strains Isolated from Wild Bird Feces

Two *E. coli* strains, PEC1011 and PEC1012, were isolated from the fecal matter of wild birds in Pakistan between 2016 and 2017. Their genome sequences were obtained by a hybrid sequencing approach, resulting in complete genome sequences. The genome of strain PEC1011 consists of one chromosome (4,872,082 bp) and one plasmid (86,434 bp). The sequencing depth of coverage was 615.5x and a total of 4,593 genes were predicted in the genome. The genome of strain PEC1012 comprises a single chromosome (4,681,476 bp) and one plasmid (77,745 bp), with a sequencing depth of coverage of 600.5x, and a total of 4,369 genes predicted in the genome (Table 1).

MLST analyses based on the Achtman scheme assigned both strains as ST2178, belonging to phylogroup B1. STs belonging to this phylogroup have been frequently isolated from animal and the environment rather than human specimens, and *E. coli* strains of the phylogroup generally have been shown to be commensals [26, 27]. ST2178 have also been frequently isolated from various animals [28-31] and remarkably, shiga-toxin producing *E. coli* (STEC) belonging to this ST was isolated from healthy cattle [32]. Furthermore, ST2178 strains have been isolated from various human specimen, with some reported as non-DEC strains and EAEC strains [33-38]. ST2178 strains have also been found in environmental samples [39] and One-health approach revealed the presence of ST2178 across the One-health sectors [40]. The virulence factors of the isolates were investigated and compared to the previously established pathotype classification of phylogroup B1 according to virulence factor repertoires [41], revealing that these isolates are presumed as non-pathogenic *E. coli* strains (Fig. S1).

In the case of ARGs in this ST, numerous studies have indicated that the presence of mobile ESBL genes such as *bla*_CTX-M_ gene as well as diverse array of ARGs in strains isolated from animals and human [29, 33, 38]. Strains PEC1011 and PEC1012 each harbored a plasmid in their genomes. The two strains exhibited differences in plasmid-borne ARG repertoires. The plasmid of strain PEC1012 (pPEC1012) carries various ARGs including *aph(6)-Id*, *aph(3'')-Ib*, *bla*_CTX-M-15_, *bla*_TEM-1_, *qnrS1*, *sul2*, *dfrA14*, *tetR*, and *tet(A)*, whereas the plasmid of strain PEC1011 (pPEC1011) harbors no ARGs (Table 1). The results underscore the importance of deciphering the plasticity of mobile ARG contents within ST2178. Although there are limited reports on this ST compared to other major STs belonging to pathogenic phylogroups, its presence in One-health sectors, pathotype reports, and mobile ARG traits of ST2187 emphasize the necessity to scrutinize the genomic characteristics of this ST using comparative genomics approach.

### Phylogenomic Analyses of *E. coli* ST2178 Strains and Their Origins

**Fig. 1:**
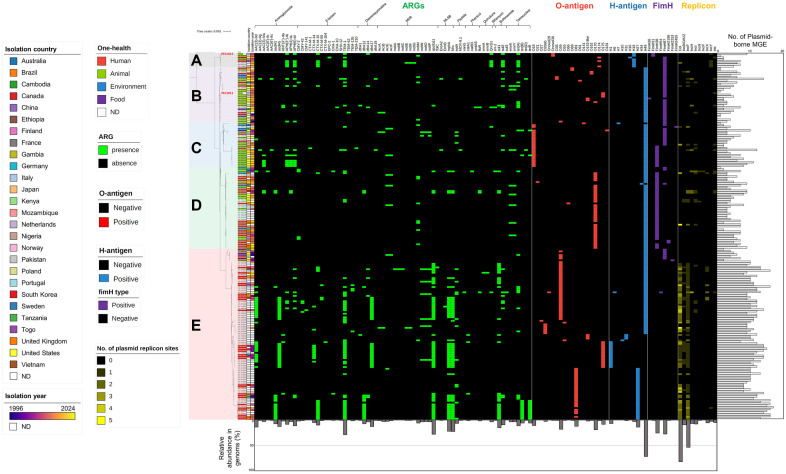
Core-genome phylogeny, One-health origins, isolation countries and years, profiles of plasmid-borne ARGs, serotypes, FimH types, plasmid replicons, and MGE abundances of 204 *E. coli* ST2178 obtained from the EnteroBase database, along with two animal isolates from this study (strains PEC1011 and PEC1012). A genome belonging to ST58 was employed as an outgroup. Distinct phylogenetic clades were delineated as subclades **A, B, C, D**, and **E**.

A total of 204 *E. coli* genomes belonging to ST2178 were retrieved from the EnteroBase database (Table S1). These genomes and two isolates’ complete genomes were used for further comparative genomics analyses. First, their phylogenetic characteristics were elucidated by core gene-based phylogenomic analyses. According to the analyses, the phylogeny of ST2178 was divided into five subclades (A, B, C, D, and E) (Fig. 1). Among these subclades, subclade E was the most prevalent, with all strains originating from human specimens, except for an early diverged branch where strains were isolated from animals and foods. On the contrary, strains belonging to subclades A and B, were frequently isolated from animals (17 out of 29 strains with isolation source information), but some strains were isolated from human specimens (8 out of 29 strains). In the case of subclade C, strains were isolated from human specimen (3 out of 16 strains with isolation source information) as well as animals (3 out of 16 strains). Environmental isolates were much less abundant than human and animal isolates, and they were located in subclades B, C, and D. Strains PEC1011 and PEC1012 were located in subclades A and B, respectively, which coincided with their animal origin (Fig. 1). The geographical origin and isolation year of the isolates were examined to prevent potential bias in subclade classification due to the presence of multiple clonal strains within each subclade. In subclade E, some strains with similar ARG repertoires across phylogenetic branches originated from different locations and years (Fig. 1), suggesting that clonal strains did not introduce bias into the classification. Additionally, the global distribution analysis of ST2178 showed that ST2178 strains were isolated in 27 countries across 6 continents, including high-income, low-income, and developing countries (Fig. 1). These findings highlight the global distribution of this minor ST.

### ARG and VF Repertoires associated with Mobile Traits in *E. coli* ST2178

**Fig. 2:**
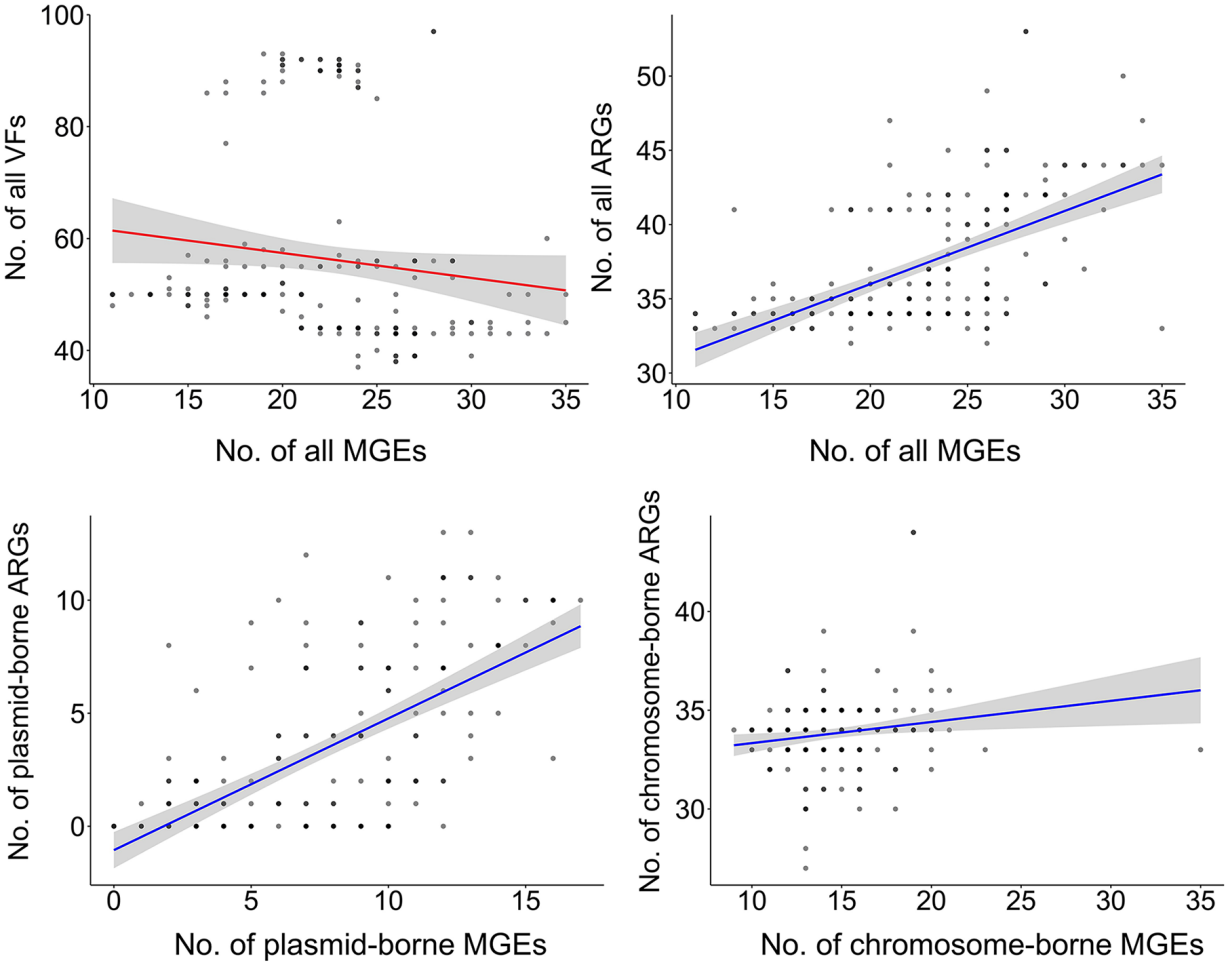
Correlation analyses between MGEs and VFs (or ARGs) in *E. coli* ST2178 strains. The number of ARGs (or VFs) and MGEs in a single genome was plotted, and linear regression was performed. Contigs of genomes were categorized as either plasmid or chromosome contigs using geNomad tool, allowing for the classification of ARGs and MGEs as either plasmid- and chromosome-borne.

To understand the potential mobilization of ARGs and VFs in ST2178, the repertoires of ARGs and VFs were surveyed, and their abundance profiles were compared to those MGEs. The amount of ARGs in ST2178 strains showed a positive correlation with the amount of MGEs (slope, 0.49; R^2^, 0.35), whereas between the amounts of VFs and MGEs, there was no correlation (slope, -0.44; R^2^, 0.02), indicating that MGEs of this ST are primarily involved in the dissemination of ARGs rather than that of VFs (Fig. 2). For the detailed comparison, ARGs were categorized as plasmid-borne and chromosome-borne based on their genetic location in plasmid and chromosome contigs, respectively. Similarly, MGEs were classified as plasmid-borne and chromosome-borne MGEs for the comparison. Plasmid-borne ARGs showed a strong positive correlation with plasmid-borne MGEs (slope, 0.58; R^2^, 0.43), whereas chromosome-borne ARGs revealed no correlation with chromosome MGEs (slope, 0.11; R^2^, 0.03) (Fig. 2). The results indicated that plasmid-borne ARGs are primarily responsible for MGE-mediated ARG dissemination through plasmids in this ST.

In line with the absence of correlation between amounts of VFs and MGEs, the profiles of VFs in ST2178 were dependent on their phylogeny. For instance, the locus of enterocyte effacement (LEE)-encoded type III secretion system (T3SS or TTSS) and adhesion genes were exclusively present in subclade D, except for the early diverged branch (Fig. S1), implying that VFs in this ST were transferred vertically rather than horizontally. It is noteworthy that strains in subclades D and E carrying extra VFs have been primarily isolated from human specimens (Fig. S1).

**Fig. 3:**
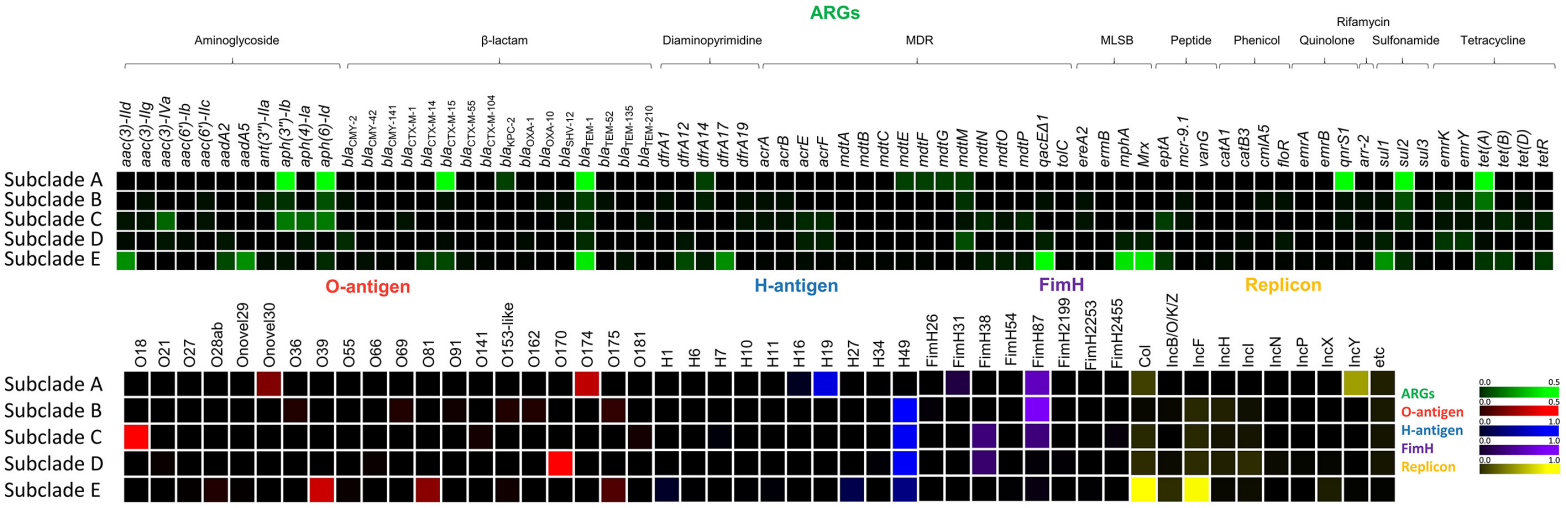
Profiles of plasmid-borne ARGs, serotypes, FimH types, and plasmid replicons in *E. coli* ST2178 subclades. The relative abundance of each feature within each subclade population is presented as a heatmap.

As demonstrated in the MGE correlation analyses, numerous chromosome-borne ARGs such as efflux pump genes and an AmpC β-lactamase gene are conserved as intrinsic resistance determinants within ST2178. Some ARGs such as *bla*_CTX-M-14_, *bla*_CTX-M-55_, *pp-cat*, *dfrA5*, *tet(B)*, and *tetR*, were identified as chromosomally acquired ARGs but their frequency was low (<5%) and their distribution was not related to subclade lineage (Fig. S2), indicating the limited implication of chromosome-borne ARGs in ARG dissemination in this ST. In contrast, plasmid-borne ARGs exhibited clear distinction according to subclade classification (Figs. 1 and 3). Strains in subclade E carried more diverse array of ARGs compared to those in other subclades. This observation correlates with a higher amount of plasmid-borne MGEs in subclade E compared to other subclades (Figs. 1 and 2). In addition, plasmid replicon sites in the genomes were analyzed, revealing that 97 genomes harbor plasmid replicon sites. Col and IncF type plasmid replicons were predominant, and these replicons were distinguishably abundant in subclade E (Figs. 1 and 3). The results suggest vital MGE-mediated mobilization through plasmids in this human-specific lineage. Aminoglycoside resistance genes (*aac(3)-IId*, *aadA2*, and *aadA5*), β-lactam resistance genes (*bla*_CTX-M-14_, *bla*_CTM-M-15_, and *bla*_TEM-1_), trimethoprim resistance genes (*dfrA12* and *dfrA17*), macrolide resistance genes (*mphA* and *mrx*), tetracycline resistance genes (*tet(B)* and *tetR*), and typical ARGs in class 1 integron (*qacED1* and *sul1*), were exclusively abundant in subclade E (Figs. 1 and 3).

**Fig. 4:**
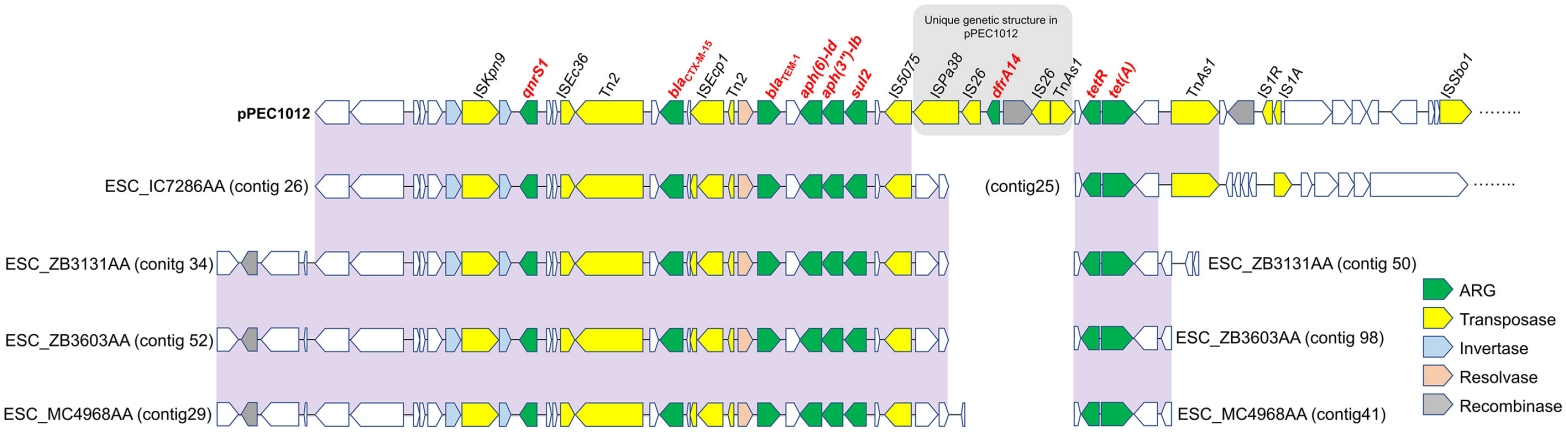
Genetic structure comparison of pPEC1012 with plasmids from strains within subclade A that harbor similar ARG repertories. ARGs and transposition-associated genes were annotated using a color code. Shades represent nucleotide sequence identity exceeding 95%. A unique insertion in plasmid pPEC1012 was indicated.

In subclades B, C, and D, ARGs were less abundant compared to those of subclade E, with no predominant ARGs identified in these subclades, along with low amounts of MGEs and plasmid replicons (Figs. 1 and 3). Including strain PEC1011 (subclade B), many strains in these subclades displayed the complete absence of plasmid-borne ARGs (Figs. 1 and 3). Subclade D, which was presumed to represent a pathotype lineage due to the presence of the extra virulence factors, showed the scarcity of ARG repertoires (Figs. 1 and 3). In contrast, subclade A, including strain PEC1012, exhibited a consensus ARG repertoires (*aph(3²)-Ib*, *aph(6)-Id*, *bla*_CTX-M-15_, *bla*_TEM-1_, *qnrS1*, *sul2*, *tetR*, and *tet(A)*), suggesting the importance of this clade as ARG transmission in this ST (Figs. 1 and 3). The gene *bla*_CTX-M-15_ was prevalent in subclades A and E as a major ESBL gene in this ST (Figs. 1 and 3). A plasmid of strain PEC1012 (pPEC1012) also harbored all of the consensus ARGs in subclade A, with the presence of *dfrA14* gene being a unique feature of the isolate (Figs. 1 and 4). Although, plasmid replicon and MGEs were less abundant in subclade A than those of subclade E, the conserved ARG repertories and the occurrence of variable genetic structures in the plasmid indicate the importance of this subclade as a potential transmission route of ARGs in *E. coli*. Furthermore, among strains in subclade A, one human isolate (Enterobase barcode No. HB9322AA; isolated at Togo in 2019) also carries the consensus ARG repertoires of pPEC1012, emphasizing the importance of this subclade.

### Serotypes and FimH Types of *E. coli* ST2178 Strains

While previous studies have reported on the serotype of *E. coli* strains belonging to ST2178, the overall landscape of serotype of this ST have not been fully elucidated. Strains in subclades A and B displayed either the absence or non-consensus distribution of O-antigen, whereas strains in subclades C and D are clearly distinguished by the presence of dominant O-antigen types, O18 and O170, respectively (Figs. 1 and 3). Subclade E strains primarily displayed O39, O81, and O175 as the major O-antigen types, with their distribution coinciding with phylogenetic branches (Figs. 1 and 3). Regarding H-antigen, subclade A strains predominantly carried mostly H19, whereas most strains in subclade B, C, and D carried H49. Subclade E exhibited three major H-antigen types: as H49, H27, and H1 (Figs. 1 and 3). The dominant serotypes of subclades C, and D, are O18:H49 and O170:H49, respectively. Subclades E carries O39:H49, O81:H1, and O175:H1 as major serotypes. No dominant serotype was observed for subclades A and B. Regarding FimH types, FimH38 and FimH87 subtypes were the dominant types in this ST. FimH38 was prevalent in subclades C and D, and FimH87 was predominant in subclades A, B, and C. In subclade E, FimH was mostly absent, except for early diverged branch, which exhibited FimH87 (Figs. 1 and 3). In the case of the isolates, strain PEC1011 was assigned to O175:H49 serotype and FimH87 type, while strain PEC1012 was classified as Onovel30:H19 and FimH31 (Fig. 1 and Table 1).

### Unique Genetic Structures in Plasmid pPEC1012

Among eight strains in subclade A, four strains carried similar ARG repertoires to those of pPEC1012. Genetic structure comparison of pPEC1012 and plasmid contigs carrying the similar ARG repertoires of four strains in subclade A, revealed a unique IS26-flanked *dfrA14* gene insertion in pPEC1012 (Fig. 4). Identical sequences of the unique insertion of pPEC1012 were found in plasmids of eight *E. coli* strains belonging to ST38, 167, 1431, and 3018 and one *Klebsiella pneumoniae* in the NCBI database. Most of them were human isolates, indicating the genetic exchange between different STs across One-health sectors. Subclade A of ST2178, particularly considering the presence of the plasmid exhibiting plasticity and its association with other STs, should be carefully surveilled.

## Conclusion

In this study, for the first time, we conducted comparative genomic analyses of *E. coli* ST2178, which have been recognized as a minor ST. A detailed phylogenetic classification at the subclade level was suggested and subclade E was identified as critical for the dissemination of antibiotic resistance and virulence in this ST. Genetic structure analysis of a plasmid of animal-originated isolate in this study highlighted the significance of subclade A in ARG dissemination. Moreover, the presence of the plasmid with expanded ARG repertoires emphasizes the urgent need for comprehensive surveillance and systematic analyses of *E. coli* isolates from developing countries. Our research provides foundational insights into phylogeny, antibiotic resistance and pathogenicity of ST2178 strains, offering a framework for future studies.

## Supplemental Materials

Supplementary data for this paper are available on-line only at http://jmb.or.kr.



## Figures and Tables

**Table 1:** Phylogenetic characteristic and plasmid information for *E. coli* isolates.

Strain	Chromosome size (bp)	Plasmid (replicon)	Plasmid size (bp)	Plasmid ARG contents	Phylogroup	Sequence type	Subclade	Serotype	FimH type
PEC1011	4,872,082	pPEC1011 (ParAB)	86,434	None	B1	2178	A	O175:H49	FimH87
PEC1012	4,681,476	pPEC1012 (IncF)	77,745	*aph(6)-Id*, *aph(3'')-Ib*, *bla*_CTX-M-15_, *bla*_TEM-1_, *qnrS1*, *sul2*, *dfrA14*, *tet(A)*	B1	2178	B	Onovel30:H19	FimH31
