# Supplementary material for: Comparative Genomic Analyses of E. coli ST2178 Strains Originated from Wild Birds in Pakistan
Source: J Microbiol Biotechnol. 2024 Aug 29;34(10):2041–8. doi: 10.4014/jmb.2407.07026 (PMC11540600; doi:10.4014/jmb.2407.07026)
Supplement: Supplementary file 1 [file jmb-34-10-2041-supple.pdf]

J. Microbiol. Biotechnol. 2024. 34(10): 2041-2048

https://doi.org/10.4014/jmb.2407.07026

Supplementary Figures

Comparative Genomic Analyses of *E. coli* ST2178 Strains Originated from Animals in Pakistan

Jung Hun Lee[1]†, Abdul Rauf Tareen[2]†, Nam-Hoon Kim[3], Chanyeong Jeong[1], Byeonghyeon Kang[1], Gwangje Lee[1], Dae-Wi Kim[3], Rabaab Zahra[2]*, and Sang Hee Lee[1]*

[1]National Leading Research Laboratory of Drug Resistance Proteomics, Department of Biological Sciences, Myongji University, Yongin 17058, Republic of Korea

[2]Department of Microbiology, Quaid-i-Azam University, Islamabad 45320, Pakistan

[3]Department of Life Sciences, Jeonbuk National University, Jeonju, 54896, Republic of Korea

*Correspondence to:

Rabaab Zahra, rzahra@qau.edu.pk, (+92) 51-9064-3228

Sang Hee Lee, sangheelee@mju.ac.kr, (+82) 031-330-6195

†These authors contributed equally to this study.

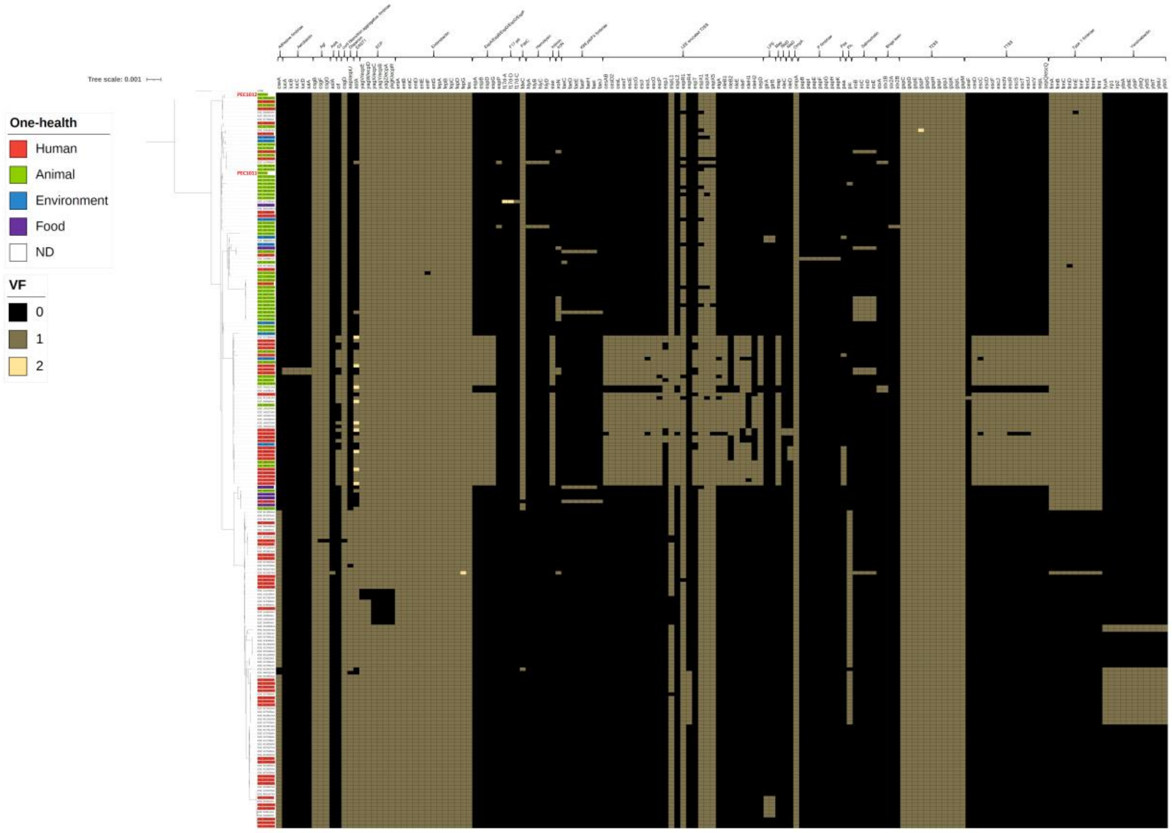

Fig. S1. Core-genome phylogeny, One health origins, and VFs of 204 E. coli ST2178 from the EnteroBase database and two animal isolates in this study (strains PEC1011 and 1012). A genome belonging to ST58 was used as an outgroup. The presence of each VF was shown with heatmap presentation.

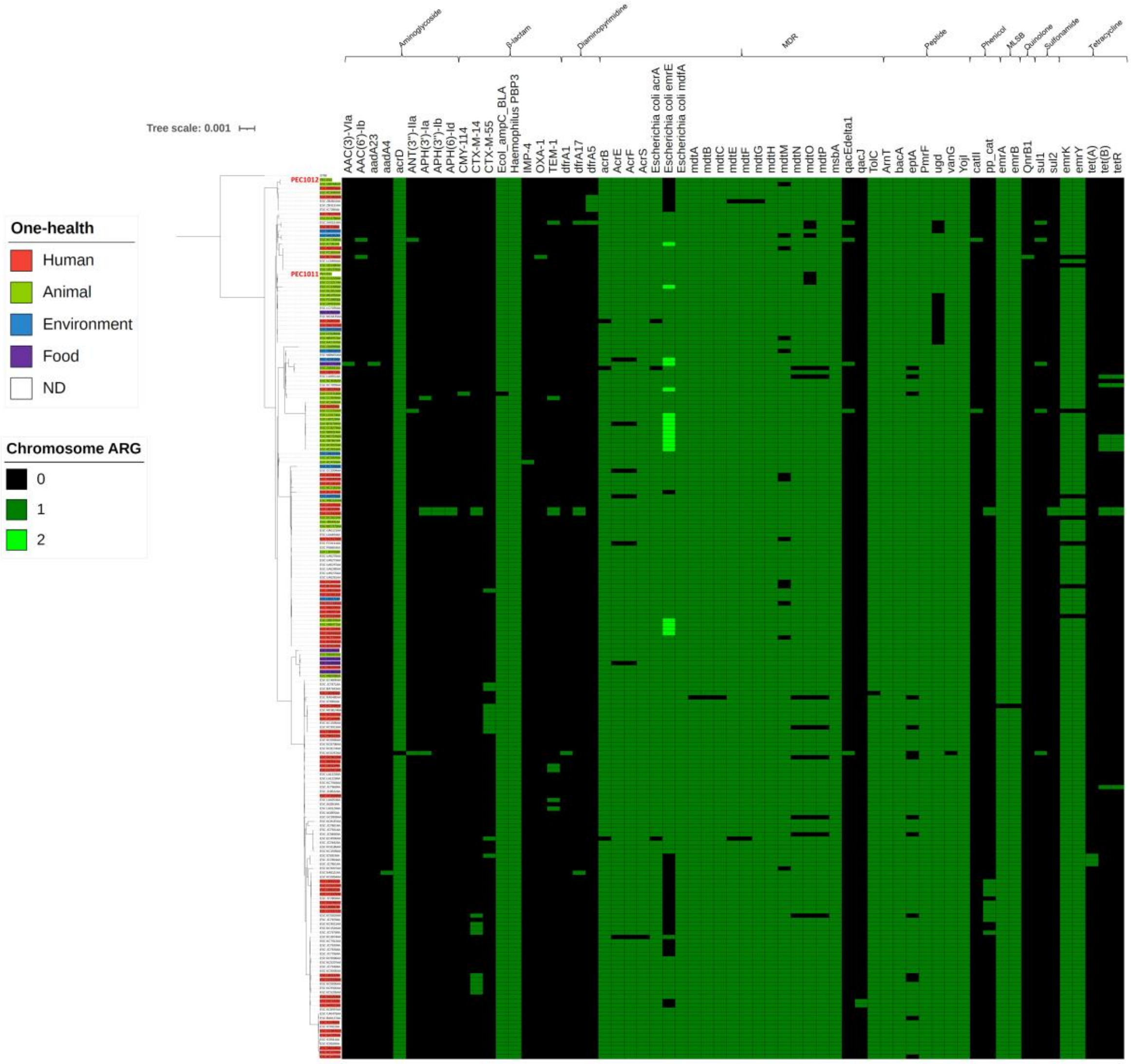

Fig. S2. Core-genome phylogeny, One health origins, and chromosome-borne ARGs of 204 E. coli ST2178 from the EnteroBase database and two animal isolates in this study (strains PEC1011 and 1012). A genome belonging to ST58 was used as an outgroup. The presence of each ARG was displayed using heatmap presentation.
